# Comprehensive UPLC-MS/MS Method for Quantifying Four Key Intestinal Permeability Markers in Caco-2 Models

**DOI:** 10.3390/molecules30173477

**Published:** 2025-08-24

**Authors:** Luciana Silva de Araújo, Eduardo José Crevelin, Luiz Alberto Beraldo de Moraes, Niege Araçari Jacometti Cardoso Furtado

**Affiliations:** 1 School of Pharmaceutical Sciences of Ribeirão Preto, University of São Paulo, Av. Prof. Dr. Zeferino Vaz, s/n, Ribeirão Preto 14040-903, SP, Brazil; luciana.araujo@usp.br; 2 Chemistry Department, School of Philosophy, Sciences and Languages, University of São Paulo, Av. Bandeirantes, 3900, Ribeirão Preto 14040-901, SP, Brazil; ejcrevelin@ffclrp.usp.br (E.J.C.); luizmoraes@ffclrp.usp.br (L.A.B.d.M.)

**Keywords:** UPLC-MS/MS, Caco-2 cell monolayer, drug absorption prediction, efflux transporter, intestinal permeability standards, P-gp Inhibition, regulatory applications, tandem mass spectrometry

## Abstract

A comprehensive UPLC-MS/MS method was developed and validated for the simultaneous separation and quantification of atenolol, propranolol, quinidine, and verapamil, using established intestinal permeability standards in the Caco-2 cell monolayer model. This in vitro model is widely accepted for predicting intestinal drug permeability and is formally recognized by global regulatory agencies, including the FDA, EMA, and WHO, as a surrogate for assessing drug permeability in biowaiver applications under the Biopharmaceutics Classification System (BCS) framework. Despite its regulatory importance, standardized methods for the simultaneous quantification of key permeability markers remain scarce. The selected compounds represent distinct transport pathways: paracellular (atenolol), passive transcellular (propranolol, verapamil), and P-glycoprotein-mediated efflux (quinidine). Method validation followed FDA guidelines and demonstrated high selectivity, linearity (r^2^ > 0.998), precision, and accuracy. Solid-phase extraction enhanced recovery and reduced matrix effects. Application to Caco-2 permeability assays confirmed expected transport profiles, including P-gp inhibition effects with verapamil. By integrating multiple analytes in a single workflow, the method improves analytical throughput, supports mechanistic interpretation, and ensures consistency across assays. This advanced separation strategy, combined with sensitive mass spectrometric detection, supports regulatory and BCS-based classification studies, contributing to the standardization of permeability assessments in drug development.

## 1. Introduction

The Caco-2 cell monolayer model is widely employed in the pharmaceutical industry as an in vitro system to evaluate intestinal drug permeability and elucidate transport mechanisms [1,2]. Its broad acceptance by major regulatory agencies, including the United States Food and Drug Administration (FDA), the European Medicines Agency (EMA), the World Health Organization (WHO), and the Brazilian Health Regulatory Agency (ANVISA), underscores its reliability and relevance for predicting in vivo drug absorption [3,4,5]. These agencies formally recognize the Caco-2 model as a regulatory-acceptable in vitro surrogate for assessing drug permeability, particularly in the context of biowaiver applications under the Biopharmaceutics Classification System (BCS) [1].

Studies have shown that Caco-2 monolayers accurately mimic drug transport across the intestinal epithelium, especially for compounds that undergo passive transcellular diffusion, which exhibits the strongest correlation with in vivo conditions [6,7]. Furthermore, the model’s capacity to simulate key gastrointestinal transport mechanisms has established it as an indispensable tool for predicting human drug absorption [7].

Among the various compounds studied using this model, atenolol is widely recognized as a reference drug for low permeability, particularly in investigations of passive transport. As a BCS Class III compound, atenolol is highly soluble but poorly permeable. Its absorption occurs via both transcellular and paracellular routes, making it a relevant standard for demonstrating the Caco-2 model’s ability to predict drug transport and absorption [7,8,9,10].

In contrast to atenolol, propranolol is recognized as a key example of high-permeability drugs, primarily absorbed through passive transcellular pathways. Propranolol has been extensively cited as a high-permeability marker in foundational studies, including the BCS framework proposed by Amidon et al. [11] and has been further emphasized in regulatory guidelines (e.g., FDA) [4,7] and comprehensive reviews such as those by Zheng and co-authors [10].

Verapamil and quinidine, while also showing high permeability, are of particular interest due to their interactions with P-glycoprotein (P-gp), a key efflux transporter involved in drug absorption [12]. Verapamil, frequently mentioned in studies such as Anderle et al. [13] and Dahlgren et al. [8], stands out as a dual-purpose marker, illustrating both high permeability and its role as a P-gp inhibitor. Similarly, quinidine highlights how active efflux can shape drug transport, as discussed in studies addressing transporter-mediated drug interactions [14].

Collectively, these compounds, atenolol, propranolol, verapamil, and quinidine, illustrate the versatility of the Caco-2 model in studying both passive diffusion and transporter-mediated mechanisms. They provide critical insights into the processes governing intestinal drug absorption [14]. However, despite their widespread use of these permeability standards, a unified, robust analytical method capable of quantifying them simultaneously has remained limited.

Several analytical methods have been developed for the determination of these compounds, each tailored to specific analytical requirements and applications. Venkatesh et al. [15] developed an RP-HPLC-UV method for the simultaneous determination of all four compounds, achieving quantification limits ranging from 0.8 to 15 μg/mL and demonstrating its applicability to rat in situ permeability studies. LC-MS/MS methods reported by Li et al. [16] and van Domburg et al. [17] focused on therapeutic drug monitoring, offering enhanced sensitivity for atenolol, propranolol, and verapamil in human plasma; however, quinidine was not included in their analytical scope. Varma et al. [18] described an HPLC-UV method for quinidine, verapamil, and propranolol, specifically designed for permeability studies, and demonstrated its suitability in rat in situ models. While these methods have successfully met their intended purposes, no approach to date has combined UPLC-MS/MS technology with complete coverage of all four regulatory-recognized permeability markers, specifically optimized and validated for Caco-2 studies.

**Figure 1:**
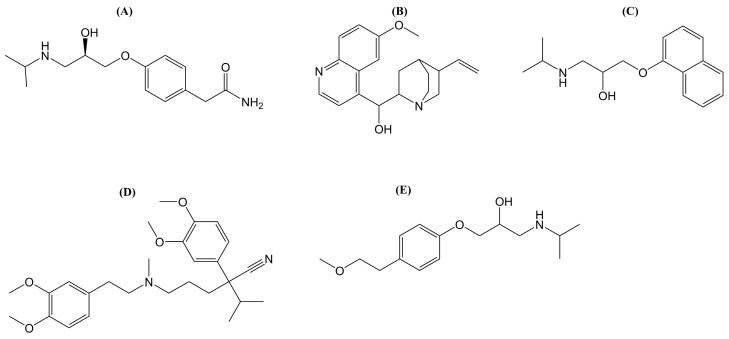
Analytical standards: (**A**) atenolol, (**B**) quinidine, (**C**) propranolol, (**D)** verapamil, and (**E**) metoprolol (internal standard—IS).

To address this gap, the present study reports the development and validation of a novel UPLC-MS/MS method for the simultaneous separation and quantification of atenolol, propranolol, quinidine, and verapamil (Figure 1), four well-established permeability reference compounds in the Caco-2 cell monolayer model. This multianalyte method, comprehensive in its inclusion of the four key regulatory-recognized permeability markers representing different intestinal transport mechanisms, enables accurate and precise quantification of compounds representing a range of permeability and transporter profiles. By allowing simultaneous measurement, the method improves analytical throughput, improves consistency, and supports more rigorous permeability assessments. These advances are particularly valuable in regulatory and biowaiver contexts, where methodological standardization and analytical robustness are paramount.

## 2. Results and Discussion

### 2.1. Bioanalytical Method Validation

#### 2.1.1. Selectivity, Linearity, Carryover, Precision and Accuracy

To ensure reliable permeability measurements in Caco-2 cells, a robust bioanalytical method is required to detect analytes across a wide dynamic range and within complex biological matrices. Our validated UPLC-MS/MS method fulfilled these criteria.

**Figure 2:**
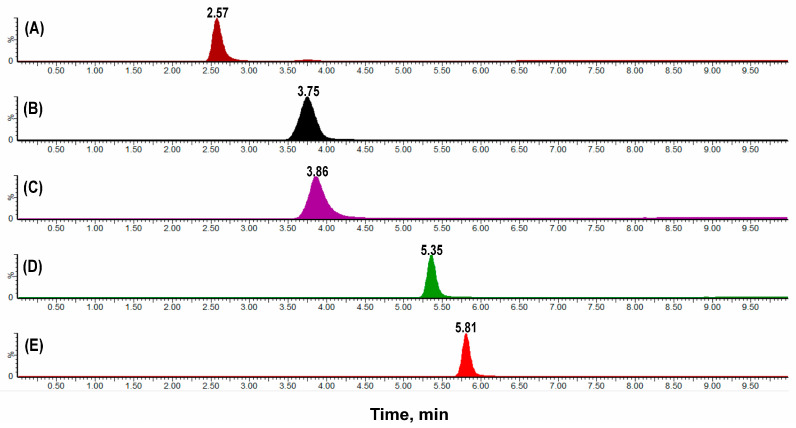
Chromatograms demonstrating the selectivity of the analytical method in the Caco-2 apical matrix spiked at the lower limit of quantification (LLOQ). Distinct peaks are shown for each standard compound: (**A**) Atenolol (t_R_ 2.57 min, 25.4 ng/mL), (**B**) Metoprolol (t_R_ 3.75 min, IS), (**C**) Quinidine (t_R_ 3.86 min, 12.7 ng/mL), (**D**) Propranolol (t_R_ 5.35 min, 25.4 ng/mL), and (**E**) Verapamil (t_R_ 5.81 min, 600 ng/mL). Each compound is clearly identified with high specificity under the established analytical conditions.

The method exhibited excellent selectivity, with no interfering peaks observed at the retention times of the analytes or the internal standard (IS) in apical and basolateral matrices, whether blank or spiked at the LLOQ. Selectivity was confirmed using blank apical and basolateral matrices, as well as matrices spiked at the lower limit of quantification (LLOQ) for each analyte (Figure 2; Figure S1, Supplementary Materials). This specificity ensures unambiguous separation and quantification of standards in permeability assays involving Caco-2 cell monolayers.

Linearity was confirmed across all analytes, with coefficients of determination ranging from 0.995 to 1.000 (Table 1). The regression residuals were normally distributed with no lack of fit (*p* > 0.05), indicating that the model is statistically robust and reliable across the entire tested concentration range.

The method demonstrated high sensitivity, with lower limits of quantification (LLOQs) meeting FDA acceptance criteria (coefficient of variation [CV] ≤ 20%; accuracy within ±20%). This sensitivity is particularly important in Caco-2 assays, where analytes may be present at low concentrations due to limited accumulation or transport, depending on their permeability characteristics.

**Figure 3:**
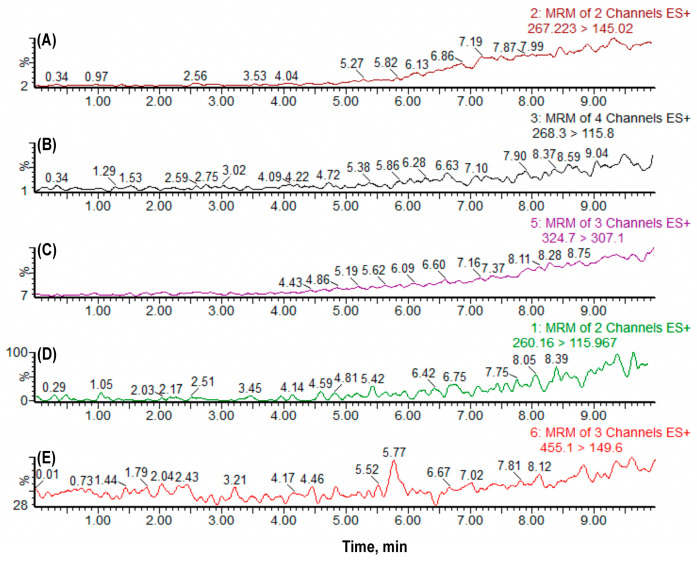
Chromatograms demonstrating the absence of carryover effect in the analytical method. The analytes (**A**) Atenolol (267.223 > 145.02), (**B**) Metoprolol (268.3 > 115.8), (**C**) Quinidine (324.7 > 307.1), (**D**) Propranolol (260.16 > 115.967), and (**E**) Verapamil (455.1 > 149.6) show no detectable residual peaks in the blank sample, confirming the absence of carryover.

No carryover was observed following high-concentration injections (Figure 3), ensuring sample-to-sample integrity, a critical requirement for multi-analyte quantification in sequential permeability assessments.

Intra- and inter-day reproducibility were confirmed, with CV% and relative error (RE%) within FDA acceptance criteria (≤15% for all concentration levels, ≤20% at LLOQ) [19] (Table 2), underscoring the method’s reliability for longitudinal studies involving multiple time points or biological replicates.

Collectively, these results validate the method’s suitability for the simultaneous separation and quantification of both low- and high-permeability markers in Caco-2 cells, enabling comprehensive modeling of passive and active transport mechanisms.

#### 2.1.2. Recovery, Matrix Effect, Stability and Robustness

Sample preparation is a common challenge in permeability studies due to the complexity of biological matrices. In this study, solid-phase extraction (SPE) was employed to improve analyte recovery and minimize matrix effects.

Extraction efficiency was consistently high, with recovery ranging from 86% to 98% (Table S1, Supplementary Materials), confirming effective analyte extraction from both apical and basolateral compartments. Internal standard-normalized matrix factors (ranging from 0.94 to 1.16) and low CVs (1.37–6.47%) indicated minimal matrix interference (Table 3).

Although some slope variability was observed for certain analytes (e.g., atenolol, verapamil; Figure S2, Supplementary Materials), this is consistent with known matrix-dependent effects. Therefore, calibration within the matrix is recommended, particularly for high-precision UPLC-MS/MS quantification.

SPE proved essential not only for improving signal clarity but also for standardizing sample preparation across varying assay conditions, an important factor in ensuring reproducibility in in vitro permeability studies. This approach supports the findings of Ötles and Kartal [20], who emphasized SPE’s ability to selectively isolate target compounds while mitigating interference from complex matrices. These advantages underscore the value of SPE as an integral component of bioanalytical workflows, particularly when accurate quantification of multiple analytes is required.

Stability assessments demonstrated consistent analyte recovery under various storage and handling conditions (Table S2, Supplementary Materials), with recoveries ranging from 85% to 99%. The method remained robust to minor variations in solvent composition (Table S3, Supplementary Materials), although deviations were observed at the LLOQ under modified H_2_O/ACN conditions. Quinidine showed a deviation of 16.23%, which is within the acceptable limit of ±20% for the LLOQ. Propranolol showed a relative error (RE%) of −32.33%, exceeding the FDA acceptance limit of ±20% by 12.33%, while atenolol showed an RE% of 20.34%, marginally exceeding the limit by 0.34%. These deviations, although notable, remained within the context of acceptable method robustness and highlight the importance of strict solvent control in analytical workflows.

Overall, the method showed excellent stability and robustness, making it suitable for bioanalytical applications that involve repeated measurements, long-term storage, or freeze–thaw cycles.

### 2.2. TEER Results and Monolayer Integrity

The integrity of the Caco-2 monolayer was monitored using transepithelial electrical resistance (TEER) throughout the 21-day differentiation protocol. TEER values stabilized at approximately 900 Ω·cm^2^ by day 9 and remained steady through day 21 (Figure S3, Supplementary Materials), confirming the formation of tight junctions and full monolayer differentiation.

Following the 4-h permeability assays, TEER values were measured again to confirm the monolayer’s structural integrity. In accordance with established guidelines and the literature recommendations [21], only monolayers with TEER values between 500 and 1100 Ω·cm^2^ before and after the assay were considered acceptable and included in the analysis, ensuring data reliability and preserving the physiological relevance of the permeability results.

Maintaining monolayer integrity is essential for distinguishing between paracellular and transcellular transport, further underscoring the importance of rigorous quality control in Caco-2-based permeability studies.

### 2.3. Cell Viability and Cytotoxicity Assessment

Lactate dehydrogenase (LDH) assays confirmed that atenolol (50 µM), verapamil (50 µM), quinidine (20 µM), and propranolol (5 µM) did not induce cytotoxicity (Figure S4, Supplementary Materials). These concentrations are thus suitable for permeability studies, preserving both monolayer integrity and cellular viability.

### 2.4. Application to Permeability Assessment and Efflux Ratio Results

**Figure 4:**
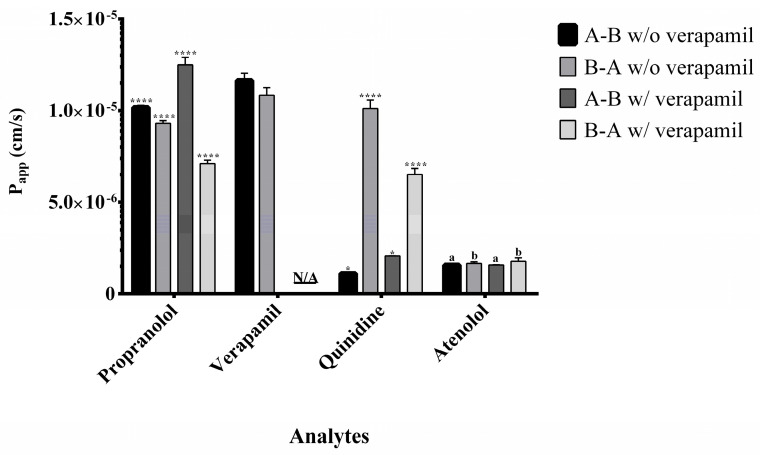
Apparent permeability (*P*app) of the analytes in the presence (w/) and absence (w/o) of verapamil in Caco-2 cell permeability assays. Experiments were conducted in both apical-to-basolateral (A→B) and basolateral-to-apical (B→A) directions. Data were analyzed using two-way ANOVA (factors: direction and treatment), followed by Tukey’s post hoc test. Pairwise comparisons were made between A→B with verapamil versus A→B without verapamil, and between B→A with versus B→A without verapamil. Biological replicates: *n* = 3. Statistical significance is indicated as **** *p* < 0.0001 and * *p* < 0.001. Identical letters denote no statistically significant differences. N/A indicates conditions where the analysis does not apply. Data are presented as mean ± standard deviation.

**Figure 5:**
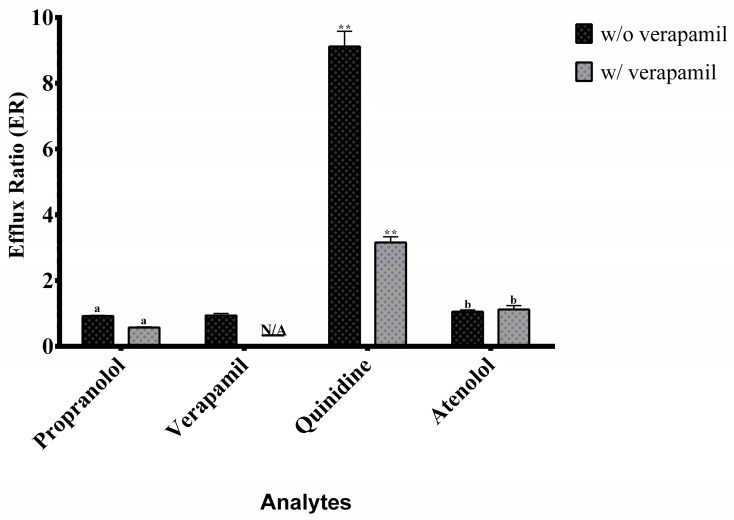
Efflux ratios of the analytes in the presence (w/) and absence (w/o) of verapamil in Caco-2 cell permeability assays. Data were analyzed using an unpaired two-tailed *t*-test to compare conditions with and without verapamil. Statistical significance is indicated as ** *p* < 0.01; ns = not significant. Identical letters indicate no statistically significant difference. N/A indicates conditions where the analysis does not apply. Data are presented as mean ± standard deviation (*n* = 3).

The bidirectional apparent permeability (*P*app) of propranolol, quinidine, and atenolol was evaluated under A→B (apical to basolateral) and B→A (basolateral to apical) conditions, both with and without the P-glycoprotein (P-gp) inhibitor verapamil. Verapamil, a well-established P-gp inhibitor, was used to assess the contribution of efflux transport to the directional permeability of these analytes and to differentiate between passive diffusion and P-gp-mediated efflux within the intestinal monolayer. Its intrinsic bidirectional permeability was also determined in the absence of an external inhibitor. Propranolol and verapamil showed high A→B and B→A *P*app values (Figure 4) with efflux ratios close to 1 (Figure 5), indicating predominant passive transcellular transport consistent with BCS Class I classification [10,22].

Quinidine displayed directional transport characteristics of active efflux via P-gp. Co-administration with verapamil increased A→B transport and reduced B→A movement, confirming P-gp inhibition and demonstrating the model’s ability to detect transporter interactions.

Atenolol consistently showed moderate permeability in both directions, unaffected by P-gp inhibition, confirming its paracellular transport route and validating its role as a moderate permeability standard [7,9].

These findings are in line with the known pharmacokinetic properties of each compound and highlight the method’s utility in differentiating between passive diffusion and transporter-mediated processes.

While many studies have focused on specific molecule transport (e.g., [23,24,25,26,27,28,29,30,31]), few have developed integrated approaches using permeability standards. Even when such standards are employed, most methods require separate assays or provide limited mechanistic insight.

Logoyda et al. [32] addressed this gap with a method for verapamil, while Venkatesh et al. [15] analyzed all four standards via RP-HPLC. However, these approaches are limited by lower sensitivity and selectivity.

In contrast, the present UPLC-MS/MS method enables the simultaneous separation and quantification of multiple standards with superior sensitivity, allowing direct comparisons across diverse transport mechanisms. This represents a significant advancement for both mechanistic investigations and regulatory applications. The inclusion of atenolol, propranolol, quinidine, and verapamil allows for a comprehensive assessment of key drug transport pathways.

By leveraging the capabilities of triple quadrupole mass spectrometry in multiple reaction monitoring (MRM) mode, originally introduced by Yost and Enke [33], this method delivers high specificity, sensitivity, and throughput. Its successful application in Caco-2 permeability assays underscores the essential role of mass spectrometry in contemporary pharmacokinetic research.

## 3. Materials and Methods

### 3.1. Reagents and Equipment

Caco-2 human colorectal adenocarcinoma cells (CRL-2102) were obtained from the Rio de Janeiro Cell Bank (BCRJ, Rio de Janeiro, Brazil). Cells were cultured in Dulbecco’s Modified Eagle Medium (DMEM) supplemented with fetal bovine serum (FBS), non-essential amino acids (NEAA) (both from Gibco™, Thermo Fisher Scientific, Grand Island, NY, USA), and penicillin-streptomycin (Sigma-Aldrich, St. Louis, MO, USA).

Trypsin (Gibco™) and phosphate-buffered saline (PBS; Gibco™) were used during routine cell maintenance. Cytotoxicity was evaluated using a lactate dehydrogenase (LDH) detection kit (Roche Applied Science, Mannheim, Germany). Dimethyl sulfoxide (DMSO, 1% *v*/*v*) and Triton X-100 (2% *v*/*v*) (both from Sigma-Aldrich) were used in cytotoxicity assays performed with a microplate spectrophotometer (Biochrom Ltd., Cambridge, UK). Hank’s Balanced Salt Solution (HBSS) containing 2-(N-morpholino) ethanesulfonic acid (MES) and N-(2-hydroxyethyl)piperazine-N′-(2- ethanesulfonic acid) (HEPES) buffers was purchased from Sigma-Aldrich. Methanol (MeOH) and acetonitrile (ACN) were obtained from Fisher Scientific (Waltham, MA, USA), and ultrapure water was generated using a Milli-Q system (MilliporeSigma, Burlington, MA, USA).

Samples from permeability assays were prepared using Discovery DSC-18 solid-phase extraction (SPE) cartridges (500 mg, 3 mL; Sigma-Aldrich). Analytical standards included atenolol, quinidine, propranolol, hydrochloride verapamil, and metoprolol (internal standard-IS), all from Sigma-Aldrich. Key equipment included Transwell^®^ plates with 1 µm polycarbonate membranes, centrifuge tubes, and cell culture plates from Corning Costar (New York, NY, USA). Samples were processed using refrigerated centrifuges (Eppendorf, Hamburg, Germany), and analyses were conducted on an Ultra-performance liquid chromatography-tandem mass spectrometry system (UPLC-MS/MS, Waters Xevo TQ-S, Milford, MA, USA) equipped with an Ascentis^®^ Express C18 column (10 cm × 4.6 mm, 2.7 μm). Monolayer integrity was monitored using an EVOM2 TEER measurement device (World Precision Instruments, Sarasota, FL, USA).

### 3.2. Instrumental Analytical Conditions

Separation and quantification of atenolol, propranolol, quinidine, and verapamil were performed using UPLC-MS/MS. Chromatographic separation employed a mobile phase consisting of (A) water with 0.1% formic acid, (B) methanol with 0.1% ammonium hydroxide, and (C) acetonitrile with 0.1% formic acid. The flow rate was maintained at 0.350 mL/min using a gradient program starting with 50% A, 45% B and 5% C, changing to 5% A, 92% B and 3% C by 6 min, maintaining these proportions until 10 min, and returning to the initial conditions by 11 min with re-equilibration until 16 min. The injection volume was set to 10 µL.

Detection was carried out in multiple reaction monitoring (MRM) mode using electrospray ionization (ESI) in positive mode. Source parameters included an interface temperature of 150 °C, a desolvation line temperature of 300 °C, and a nebulizer gas flow of 7.00 and 6.68 Bar. These settings were optimized to achieve precise and reproducible analyte quantification. Specific MRM transitions and MS parameters are provided in the supplementary data (Table S4).

### 3.3. Bioanalytical Method Validation

The bioanalytical method used to separate and quantify atenolol, propranolol, quinidine, and verapamil in samples from intestinal permeability assays was validated in compliance with the FDA Bioanalytical Method Validation Guidance for Industry [19]. The validation process involved evaluating selectivity, linearity, precision, accuracy, recovery, matrix effects, stability, and robustness. All steps were conducted using metoprolol as the internal standard and were performed in both the apical and basolateral Caco-2 matrices to ensure reliability and relevance to intestinal permeability experimental conditions. Full validation was carried out in the basolateral matrix, which represents the most complex condition due to the presence of fetal bovine serum. To demonstrate matrix equivalence and ensure the applicability of the method in both compartments, all parameters were confirmed in the apical matrix using a reduced number of replicates. Validation followed the FDA bioanalytical method validation guidelines and included: selectivity assessment using blank matrices; linearity evaluation with calibration curves (6–8 points in duplicate, over three days); precision and accuracy assessments using four QC levels (LLOQ, low, medium, high), with five replicates for intra-day and three replicates over three days for inter-day precision; recovery at three concentrations, each in triplicate; matrix effect evaluation at two QC levels; carryover analysis; stability testing under multiple conditions; and robustness testing under varied analytical conditions.

#### 3.3.1. Selectivity

Selectivity was assessed by analyzing blank matrix samples, matrix-spiked samples, and solvent standards. Blank matrix samples (Caco-2 apical and basolateral matrices free of analytes) were processed through solid-phase extraction to confirm the absence of interfering peaks at the retention times of the analytes and internal standard. Matrix-spiked samples consisted of blank matrices fortified with analytes at LLOQ concentrations (atenolol 25.4 ng/mL, quinidine 12.7 ng/mL, propranolol 25.4 ng/mL, verapamil 600 ng/mL) and metoprolol (200 ng/mL) as internal standard, also subjected to extraction. Solvent standards, prepared in methanol at identical concentrations, bypassed the extraction step.

Three replicate samples of each type and matrix were analyzed, reflecting the standardized composition of commercially sourced Caco-2 matrices. Acceptance criteria required blank sample responses to be less than 20% of the LLOQ response for analytes and less than 5% for the internal standard, in accordance with FDA bioanalytical method validation guidelines [19].

#### 3.3.2. Linearity

Calibration curves were prepared by spiking blank Caco-2 matrices with mixed standard solutions containing atenolol (25.4–600 ng/mL), propranolol (25.4–600 ng/mL), quinidine (12.7–406.3 ng/mL), and verapamil (600–6500 ng/mL), covering the expected dynamic range for intestinal permeability studies. Two separate calibration curves were constructed to account for differences in concentration ranges: one for atenolol, propranolol, and quinidine, and another for verapamil. Metoprolol was used as the internal standard at 200 ng/mL for the first curve and 1200 ng/mL for the verapamil curve. Calibration standards were prepared by spiking blank matrices with the working solutions, and all samples underwent solid-phase extraction prior to LC-MS/MS analysis. Linearity was assessed by least squares regression, along with evaluation of lack of fit, normality of residuals, and coefficients of determination (R^2^). Linearity was considered acceptable when the calibration curve demonstrated a coefficient of determination (R^2^) ≥ 0.99 and individual back-calculated concentrations were within ±15% of nominal values (±20% at the LLOQ). In accordance with FDA bioanalytical method validation guidelines, at least 75% of calibration points were required to meet these criteria to ensure overall curve validity.

#### 3.3.3. Carryover Analysis

Carryover analysis was performed in accordance with FDA guidelines to verify the absence of significant residual analyte interference between injections. High concentration samples at the upper limit of quantification (ULOQ) were injected for atenolol (600 ng/mL), quinidine (406.3 ng/mL), propranolol (600 ng/mL), and verapamil (6500 ng/mL), followed directly by blank samples. The blank samples were evaluated to ensure that residual signals remained below the acceptable thresholds. Carryover was considered acceptable if the analyte response in the blank did not exceed 20% of the response at the lower limit of quantification (LLOQ) for each compound, and 5% of the response for the internal standard, as recommended by the FDA bioanalytical method validation guidance [19].

#### 3.3.4. Precision and Accuracy

Precision and accuracy were evaluated using quality control (QC) samples prepared at four concentration levels: LLOQ, low, medium, and high. QC samples were prepared primarily in Caco-2 basolateral matrix (containing fetal bovine serum, representing the most complex analytical condition), with confirmation studies conducted in apical matrix. The concentrations used were 25.4 ng/mL (LLOQ), 76.2 ng/mL (low), 312.7 ng/mL (medium), and 450 ng/mL (high) for atenolol and propranolol; 12.7 ng/mL (LLOQ), 38.1 ng/mL (low), 209.5 ng/mL (medium), and 304.73 ng/mL (high) for quinidine; and 600 ng/mL (LLOQ), 900 ng/mL (low), 3550 ng/mL (medium), and 4875 ng/mL (high) for verapamil. QC samples were prepared separately from calibration standards by spiking blank matrices with analyte solutions, ensuring independent assessment of method performance. Intra-assay precision was assessed by analyzing QC samples within the same analytical run, while inter-assay precision was determined across multiple runs. Accuracy was evaluated by comparing the measured concentrations to their nominal values, ensuring adherence to predefined acceptance criteria. According to FDA bioanalytical method validation guidelines, accuracy was considered acceptable when the mean value was within ±15% of the nominal concentration (±20% for LLOQ), and precision was acceptable when the coefficient of variation (CV) did not exceed 15% (20% for LLOQ).

#### 3.3.5. Recovery and Matrix Effects

Recovery was determined by comparing the peak area of samples spiked before extraction (pre-extraction) with those spiked after extraction (post-extraction) at equivalent concentrations. The evaluation was performed at three QC levels: 76.2 ng/mL (low), 312.7 ng/mL (medium), and 450 ng/mL (high) for atenolol and propranolol; 38.1 ng/mL (low), 209.5 ng/mL (medium), and 304.7 ng/mL (high) for quinidine; and 900 ng/mL (low), 3550 ng/mL (medium), and 4875 ng/mL (high) for verapamil, using six replicates per level. All samples were prepared in the Caco-2 basolateral matrix. Recovery (%) was calculated as shown in Formula (1):

(1) $$Recovery\left(\%\right)=\frac{Peak\;area\;of\;pre-extraction\;spiked\;sample}{Peak\;area\;of\;the\;post-extraction\;spiked\;sample}\;\times 100$$

Matrix effects were assessed using the internal standard-normalized matrix factor (IS-normalized MF), as recommended by the FDA. The matrix factor (MF) (Formula (2)) was calculated for each analyte and its respective internal standard in both apical and basolateral matrices at two QC levels (low and high), using six replicates per matrix. The IS-normalized MF was obtained by dividing the MF of the analyte by the MF of the internal standard.

(2) $$MF=\frac{Peak\;area\;in\;post-extraction\;spiked\;sample}{Peak\;area\;in\;neat\;solution}$$

The coefficient of variation (CV%) of the IS-normalized MF across replicates was used to assess matrix effect precision, with acceptable variability defined as CV ≤ 15%, in accordance with FDA bioanalytical method validation guidelines [19].

#### 3.3.6. Stability

Stability studies were conducted to evaluate the behavior of atenolol, quinidine, propranolol, and verapamil under different conditions. These tests included assessing short-term stability at room temperature, long-term stability during storage at −20 °C for up to 45 days, and stability across three freeze-thaw cycles. Additionally, the stability of the analytes was tested in the media used for intestinal permeability assays, specifically HBSS with MES and HEPES buffers at pH 6.0 and 7.4.

#### 3.3.7. Robustness

Robustness was evaluated by introducing small, deliberate variations in analytical parameters, such as flow rate of the mobile phase (±2%), column temperature (±3 °C), and mobile phase pH (±0.05 units). These tests ensured that the method’s performance was unaffected by minor operational deviations.

### 3.4. Cytotoxicity Assays

Cytotoxicity was assessed using the LDH release assay adapted from Li et al. [34,35]. In summary, LDH released into the extracellular environment catalyzes the reduction of NAD^+^ to NADH and H^+^ through the oxidation of lactate to pyruvate. Subsequently, a catalyst (diaphorase) transfers H/H^+^ from NADH + H^+^ to a tetrazolium salt (iodonitrotetrazolium, INT), resulting in the formation of a red-colored formazan product [36]. Test compounds were dissolved in DMSO at a final concentration of 1% (*v*/*v*) in HBSS with 10 mM MES buffer (pH 6.0). Triton X-100 (2% *v*/*v*) served as a positive control to induce complete cell lysis. The cells were incubated with the test solutions for 4 h at 37 °C, and LDH release into the supernatant was measured using a commercial LDH detection kit, following the manufacturer’s instructions. Absorbance was recorded at 490 nm using a microplate spectrophotometer. Cytotoxicity was calculated as a percentage of total LDH release using the Formula (3) [35]:

(3) $$Cytotoxicity\left(\%\right)=\frac{Sample\;LDH\;Release-Background}{Total\;LDH\;Release-Background}\times 100$$

All experiments were conducted in triplicate, with the results expressed as mean values accompanied by standard deviations.

### 3.5. Caco-2 Cell Permeability Assay

Caco-2 cells were cultured in DMEM supplemented with 10% FBS, 1% NEAA, and antibiotics. The cells were seeded at a density of 1 × 10^5^ cells/cm^2^ onto 1 µm polycarbonate inserts (Transwell^®^, 6-well format, Corning Inc., Corning, NY, USA) and incubated at 37 °C in a humidified atmosphere with 10% CO_2_/90% O_2_. Over 21 days, the cells differentiated into a confluent monolayer. The Transepithelial Electrical Resistance (TEER) was determined using the Formula (4) [37]:

TEER = (R_total_ − R_blank_) × *A* (4)

In this formula, R_total_ represents the electrical resistance measured across the cell monolayer and the insert (Ω), while R_blank_ corresponds to the resistance of an insert without cells (Ω). The term *A* refers to the surface area of the membrane (cm^2^). TEER values were expressed in Ω⋅cm^2^ [37].

TEER was monitored throughout the differentiation process and measured again immediately before and after the permeability assays to confirm the integrity of the monolayers. Only monolayers with TEER values exceeding 500 Ω⋅cm^2^ were included in the experiments [21,38]. For the permeability assays, the apical compartment, simulating the intestinal lumen, was filled with HBSS containing 10 mM MES at pH 6.0, while the basolateral compartment, representing systemic circulation, contained HBSS with 10 mM HEPES and 4% FBS at pH 7.4. Test compounds were added individually to the apical compartment of separate Transwell inserts at the following concentrations: atenolol (50 µM), propranolol (5 µM), quinidine (20 µM) and verapamil (50 µM). Their transport to the basolateral compartment was monitored. Bidirectional assays were conducted to evaluate passive diffusion and active transport mechanisms.

The experiments were performed in the presence and absence of verapamil, a P-glycoprotein inhibitor, at a final concentration of 50 µM. Verapamil was added to both compartments, and the monolayers were pre-incubated for 30 min. After this pre-incubation, verapamil was removed, and the test compounds were added to the apical compartment along with verapamil at a concentration of 50 µM. This setup allowed the evaluation of the influence of P-glycoprotein-mediated efflux on the transport of the test compounds [14].

The apparent permeability coefficient (*P*app) was calculated using Formula (5) [39]:

*P*app = ΔC/Δt × *A* × *C*_0_ (5)

In this equation, ∆C/∆t represents the rate of compound transport (µg/s), *A* is the membrane surface area (cm^2^), and *C*_0_ is the initial concentration in the donor compartment (µg/cm^3^).

The efflux ratio (*ER*) was determined using the Formula (6) [14]:

*ER* = *P*app (B → A)/*P*app (A → B) (6)

In this formula, *P*app (B→A) and *P*app (A→B) are the permeability coefficients for the basolateral-to-apical and apical-to-basolateral directions, respectively. Compound concentrations in the apical, basolateral, and cellular compartments were determined using UPLC-MS/MS.

### 3.6. Preparation of Samples

During method development, protein precipitation (PPT) approaches using acetonitrile and methanol were initially evaluated. However, PPT with acetonitrile resulted in poor atenolol recovery due to its low solubility in the organic solvent-rich supernatant. Methanol-based PPT improved atenolol solubility but led to less efficient protein precipitation, producing turbid extracts unsuitable for direct UPLC–MS/MS analysis. Consequently, solid-phase extraction (SPE) was selected as the optimal sample preparation technique, as it effectively removed matrix interferences.

Samples from the apical and basolateral compartments were processed using SPE to isolate and concentrate the analytes. Sample pH was adjusted to 11 to optimize analyte retention based on pKa compatibility, and the samples were loaded onto SPE cartridges preconditioned with 3 mL each of methanol, Milli-Q water, and basified Milli-Q water (pH 11). After loading, the cartridges were washed with 3 mL of basified Milli-Q water to remove impurities, and analytes were eluted with 9 mL of methanol. The methanolic eluates were evaporated to dryness using a rotary evaporator at 40 °C, and the residues were reconstituted in methanol. The reconstituted samples were then transferred to vials and prepared for UPLC–MS/MS analysis.

The inserts used for the permeability assays were prepared by adding glass beads to disrupt the cellular monolayer, aiding in the extraction of analytes. Methanol was added to the samples, followed by vortexing to initiate cell lysis. To further enhance the disruption caused by the glass beads, the samples were subjected to ultrasonication for 30 min. Subsequently, the samples were centrifuged at 4 °C for 15 min at 5000 RPM in a refrigerated centrifuge to separate the supernatant, which was carefully collected for analysis.

To facilitate the understanding of this process, a detailed workflow is provided as a flowchart in the supplementary data (Figure S5), outlining the integration of these steps for sample preparation.

### 3.7. Statistical Analyses

Statistical analyses were conducted to thoroughly evaluate the experimental data. Multifactorial comparisons were assessed using two-way ANOVA, while one-way ANOVA followed by Tukey’s post hoc test was employed for single-factor analyses. Statistical significance was set at *p* < 0.05, and all results are expressed as mean ± standard deviation (SD). Additional details regarding the statistical methods and analyses can be found in the captions accompanying each figure and table.

## 4. Conclusions

This study presents a validated UPLC-MS/MS method for the simultaneous separation and quantification of atenolol, propranolol, quinidine, and verapamil, offering a practical and reliable solution for drug permeability studies. By addressing diverse permeability profiles in a single workflow, this method simplifies the analytical process and provides consistent results, ensuring compliance with regulatory standards.

The method addresses current limitations in the field, including fragmented assay workflows, limited sensitivity, and inconsistent use of permeability standards. Its application can enhance drug classification under the BCS framework and support regulatory submissions by ensuring model integrity and analytical robustness.

## Figures and Tables

**Table 1:** Parameters assessed to confirm the linearity of the method, including the limits of detection (LOD) and quantification (LLOQ).

Analyte	Regression Equation	Coefficients of Determination	Lack of Fit (*p*-Value)	Normality (*p*-Value)	LOD/LLOQ * (ng/mL)
Atenolol	*Y* = 4.7817*x* − 0.0809	0.995	0.091	0.790	10/25.4
Quinidine	*Y* = 4.2705*x* − 0.00861	0.999	0.766	0.049	2/12.7
Propranolol	*Y* = 2.4184*x* − 0.04191	0.999	0.334	0.678	1/25.4
Verapamil	*Y* = 5.498*x* + 8.632	1.000	0.069	0.081	1/600

* Limit of detection (LOD) and limit of quantification (LLOQ) values refer to method limits determined in the Caco-2 basolateral matrix, including all sample preparation steps. The working ranges of the calibration curves are described in Section 3.3.2 (Linearity).

**Table 2:** Precision and accuracy results for the method.

Analyte	Level	Intra-Day Precision (CV%)			Inter-Day Precision (CV%)	Intra-Day Accuracy (RE%)			Inter-Day Accuracy (RE%)
		Day 1	Day 2	Day 3		Day 1	Day 2	Day 3	
Atenolol	LLOQ	4.87	7.34	1.61	5.26	−0.53	−13.10	−16.69	−19.44
	Low	1.65	1.92	15.10	2.58	−6.91	6.69	−6.20	−8.33
	Mid	2.87	0.42	12.95	3.53	4.98	1.10	−4.34	11.80
	High	2.37	0.31	15.08	1.62	−3.26	−0.62	−9.51	−14.82
Quinidine	LLOQ	1.86	4.00	0.42	1.05	0.02	−14.13	15.93	−14.41
	Low	1.19	0.71	0.84	3.56	−0.44	7.80	12.17	−4.78
	Mid	1.31	0.24	3.55	1.67	3.92	−2.21	2.17	0.24
	High	0.73	0.67	1.39	1.19	−13.22	−13.97	−1.94	−6.40
Propranolol	LLOQ	6.59	6.97	9.81	2.26	−5.27	−17.41	−8.50	−4.58
	Low	3.09	1.75	8.87	2.20	−2.27	7.00	−3.54	1.21
	Mid	4.01	0.07	13.41	4.09	5.07	−2.21	−10.62	1.44
	High	0.34	0.96	13.01	7.25	−1.38	−11.32	−8.21	11.62
Verapamil	LLOQ	1.55	7.23	1.73	4.89	−4.07	0.94	9.51	15.69
	Low	6.42	1.29	3.36	0.39	0.50	5.95	−6.18	4.82
	Mid	0.34	1.94	0.71	0.63	0.86	0.04	−8.07	−8.53
	High	1.73	0.57	4.56	3.69	5.52	7.61	−4.25	2.12

CV% = Coefficient of Variation (indicates precision); RE% = Relative Error (indicates accuracy). QC Levels: LLOQ = Lower Limit of Quantification; Low QC = Low Quality Control; Mid QC = Medium Quality Control; High QC = High Quality Control. The actual concentrations for LLOQ, low, mid, and high QC levels are detailed in Section 3.3.4 (Precision and Accuracy).

**Table 3:** Matrix effect assessment using the internal standard-normalized matrix factor (IS-Normalized MF).

Analyte	Level	IS-Normalized MF (*n* = 6)	Mean IS-Normalized MF	CV (%)
Atenolol	Low	0.98, 0.89, 0.92, 1.04, 0.89, 0.91	0.94	6.47
	High	1.04, 1.06, 1.04, 1.04, 1.06, 1.08	1.05	1.37
Quinidine	Low	0.99, 1.03, 0.96, 1.06, 0.96, 0.96	0.99	4.55
	High	1.05, 1.05, 1.07, 1.08, 1.08, 1.07	1.07	1.38
Propranolol	Low	0.98, 0.93, 0.89, 1.02, 0.86, 0.95	0.94	6.02
	High	1.20, 1.13, 1.14, 1.17, 1.21, 1.12	1.16	3.20
Verapamil	Low	0.90, 0.90, 0.91, 1.04, 0.91, 0.96	0.94	6.14
	High	0.97, 1.11, 1.13, 0.97, 1.11, 1.13	1.07	7.32

Notes: Precision is expressed as the coefficient of variation (CV%). IS-Normalized MF represents the internal standard-normalized matrix factor. Low and High QC concentrations: atenolol and propranolol (76.2 and 450 ng/mL), quinidine (38.1 and 304.73 ng/mL), and verapamil (900 and 4875 ng/mL).

## Data Availability

The original contributions presented in this study are included in the article/Supplementary Materials. Further inquiries can be directed to the corresponding author.

## Supplementary Materials

The following supporting information can be downloaded at: https://www.mdpi.com/article/10.3390/molecules30173477/s1, Table S1: Solid-phase extraction (SPE) recovery of the analytes; Table S2: Recovery values (%) of the analytes under various stability conditions; Table S3: Parameters used to assess the robustness of the method; Table S4: Optimized mass spectrometer parameters for analyte quantification. Highlighted are the ions selected for quantification; Figure S1: Chromatograms for the selectivity assessment of the UPLC–MS/MS method. Representative MRM chromatograms are shown for: the blank apical matrix (left column), the blank basolateral matrix (middle column), and the basolateral matrix spiked at the lower limit of quantification (LLOQ) for each analyte (right column). The rows correspond to: (A) Atenolol; (B) internal standard, Metoprolol; (C) Quinidine; (D) Propranolol and (E) Verapamil; Figure S2: Comparison of calibration curves prepared in methanol and matrix for atenolol, propranolol, quinidine, and verapamil; Figure S3: Transepithelial electrical resistance (TEER) measurements of Caco-2 cells cultured on Transwell™ permeable supports for 21 days. Results are expressed as mean ± standard deviation (SD). Data were analyzed using one-way analysis of variance (ANOVA) followed by Tukey’s post hoc test. Values marked with * indicate statistically significant differences according to Tukey’s test (*p* < 0.05); Figure S4: Cytotoxicity analysis of different compounds in Caco-2 cell culture. (A) atenolol (A50 and A100 µM), (B) quinidine (Q10, Q20, and Q30 µM), (C) propranolol (P5 and P10 µM), and (D) verapamil (V25, V50, and V100 µM). Triton X-100 (2%) was used as the positive control. Statistical significance: #### *p* < 0.0001 vs. basal control; **** *p* < 0.0001 vs. positive control (Triton X-100). HBSS with 1% DMSO was used as the basal control; Figure S5: Workflow diagram illustrating the step-by-step procedure for sample preparation.

## Abbreviations

The following abbreviations are used in this manuscript:

ACN	Acetonitrile
ANOVA	Analysis of Variance
A→B	Apical to Basolateral
B→A	Basolateral to Apical
BCS	Biopharmaceutics Classification System
CAPES	Coordination for the Improvement of Higher Education Personnel
CV	Coefficient of Variation
DMEM	Dulbecco’s Modified Eagle Medium
DMSO	Dimethyl Sulfoxide
EMA	European Medicines Agency
ESI	Electrospray Ionization
ER	Efflux Ratio
FAPESP	São Paulo Research Foundation
FBS	Fetal Bovine Serum
FDA	Food and Drug Administration
HBSS	Hank’s Balanced Salt Solution
HEPES	N-(2-hydroxyethyl)piperazine-N′-(2-ethanesulfonic acid)
INT	Iodonitrotetrazolium
IS	Internal Standard
LDH	Lactate Dehydrogenase
LOD	Limit of Detection
LLOQ	Lower Limit of Quantification
MF	Matrix Factor
MES	2-(N-morpholino)ethanesulfonic acid
MRM	Multiple Reaction Monitoring
NEAA	Non-Essential Amino Acids
N/A	Not Applicable
PBS	Phosphate-Buffered Saline
*P*app	Apparent Permeability Coefficient
P-gp	P-glycoprotein
QC	Quality Control
RE	Relative Error
SPE	Solid-Phase Extraction
TEER	Transepithelial Electrical Resistance
ULOQ	Upper Limit of Quantification
UPLC-MS/MS	Ultra-Performance Liquid Chromatography-Tandem Mass Spectrometry
WHO	World Health Organization
