# Supplementary material for: Comprehensive UPLC-MS/MS Method for Quantifying Four Key Intestinal Permeability Markers in Caco-2 Models
Source: Molecules. 2025 Aug 24;30(17):3477. doi: 10.3390/molecules30173477 (PMC12430079; doi:10.3390/molecules30173477)
Supplement: Supplementary file 1 [file molecules-30-03477-s001.zip › molecules-3808506-supplementary.pdf]

Supplementary Materials

Comprehensive UPLC-MS/MS Method for Quantifying Four Key Intestinal Permeability Markers in Caco-2 Models

Luciana Silva de Araújo [1], Eduardo José Crevelin [2], Luiz Alberto Beraldo de Moraes [2] and Niege Araçari Jacometti Cardoso Furtado [1,*]

[1] School of Pharmaceutical Sciences of Ribeirão Preto, University of São Paulo, Av. Prof. Dr. Zeferino Vaz, s/n, Ribeirão Preto 14040-903, SP, Brazil
[2] Chemistry Department, School of Philosophy, Sciences and Languages, University of São Paulo,
Av. Bandeirantes, 3900, Ribeirão Preto 14040-901, SP, Brazil
* Correspondence: niege@fcfrp.usp.br; Tel.: +55-16- 33154305

Table S1. Solid-phase extraction (SPE) recovery of the analytes.

Analyte	Level	Apical Recovery (%)	Basolateral Recovery (%)
Atenolol	Low	95.74 ± 3.46	97.12 ± 10.46
	Medium	92.91 ± 8.07	98.04 ± 9.72
	High	94.69 ± 4.12	93.12 ± 4.57
Quinidine	Low	89.74 ± 1.40	90.65 ± 11.94
	Medium	91.68 ± 1.66	91.74 ± 12.18
	High	91.99 ± 3.32	87.03 ± 4.65
Propranolol	Low	88.34 ± 5.90	91.27 ± 5.06
	Medium	90.39 ± 2.26	91.91 ± 8.02
	High	90.27 ± 8.66	89.65 ± 5.67
Verapamil	Low	85.99 ± 15.86	97.41 ± 7.02
	Medium	97.62 ± 12.97	95.84 ± 13.57
	High	87.50 ± 11.47	89.12 ± 6.31

Notes: The data are reported as mean ± standard deviation.

Table S2. Recovery values (%) of the analytes under various stability conditions.

Condition	Level	Atenolol	Quinidine	Propranolol	Verapamil
	LLOQ	89 ± 5.06	90.1 ± 2.0	89.12 ± 6.21	90.12 ± 2.3
HBSS + FBS + HEPES pH 7.4	Medium	92.65 ± 6.2	94.65 ± 1.1	89.99 ± 0.8	90.78 ± 1.5
	High	95.14 ± 4.23	96.0 ± 1.34	95.45 ± 1.2	98.64 ± 1.6
	LLOQ	87.2 ± 3.21	84.8 ± 3.2	85.21 ± 2.7	86.14 ± 6.2
HBSS + MES pH 6.0	Medium	94.65 ± 4.87	86.54 ± 6.21	88.23 ± 5.6	88.97 ± 5.12
	High	94.68 ± 3.6	87.65 ± 6.32	85.21 ± 6.3	88.69 ± 5.17
	LLOQ	85.5 ± 2.8	87.52 ± 6.30	86.45 ± 21	86.98 ± 6.12
Freeze-Thaw (3 cycles)	Medium	96.65 ± 4.12	98.65 ± 5.5	97.10 ± 6.3	91.10 ± 1.34
	High	93.12 ± 4.6	89.65 ± 5.6	94.65 ± 2.3	90.21 ± 1.29
	LLOQ	86.2 ± 5.12	86.21 ± 1.2	87.65 ± 5.12	87.94 ± 6.32
Long-Term (36d)	Medium	89.0 ± 6.32	89.99 ± 1.21	87.54 ± 1.03	87.0 ± 0.4
	High	89.9 ± 4.12	91.5 ± 12.5	89.6 ± 1.8	86.34 ± 1.67

Table S3. Parameters used to assess the robustness of the method.

Analyte	Level	Original Method		Temperature (Δ3°C)		MeOH (Δ2%)		H_2O/ACN (Δ0.05 F.A.)	
		Precision (CV%)	RE (%)	Precision (CV%)	RE (%)	Precision (CV%)	RE (%)	Precision (CV%)	RE (%)
Atenolol	LLOQ	3.49	18.09	0.52	17.43	1.72	14.50	0.44	20.34
	Mid	1.57	9.93	0.89	8.99	1.51	6.25	0.73	12.54
	High	0.73	10.61	1.55	10.27	0.56	8.46	0.50	12.42
Quinidine	LLOQ	2.29	7.80	0.81	10.05	1.67	10.00	16.23	-4.60
	Mid	1.07	2.55	1.04	7.98	0.99	6.74	0.57	15.45
	High	0.76	2.38	1.67	5.66	1.47	8.22	1.03	15.78
Propranolol	LLOQ	0.61	-2.39	0.98	-5.79	1.06	-2.34	6.10	-32.33
	Mid	1.38	-2.71	1.38	-6.26	1.46	-1.99	4.57	-13.32
	High	0.67	-2.52	3.03	-9.82	1.10	-2.17	1.71	-7.52
Verapamil	LLOQ	3.62	13.95	2.15	13.91	4.98	14.35	11.49	12.03
	Mid	1.64	-0.94	1.66	-4.05	3.20	-0.98	4.30	8.73
	High	1.12	-0.19	1.03	-2.76	0.54	-1.98	1.82	9.37

Notes: Notes: F.A. = formic acid. Precision is expressed as coefficient of variation (CV%), and accuracy is presented as relative error percentage (RE%).

Table S4. Optimized mass spectrometer parameters for analyte quantification. Highlighted are the ions selected for quantification.

Compound	Ionization Mode (ESI)	MRM Transition (Precursor ion > Product ion)	Collision Energy (eV)	Capillary Voltage (kV)	Cone Voltage (V)
Atenolol	+	**267.22 > 145.02**	20	3.40	36
		267.22 > 190.07	16	3.54	
Metoprolol	+	268.30 > 73.70	30	3.40	40
		**268.30 > 115.80**	25	3.54	
Quinidine	+	324.70 > 252.90	20	3.40	40
		**324.70 > 307.10**	20	3.54	
Propranolol	+	**260.16 > 115.97**	14	3.40	86
		260.16 > 182.94	14	3.54	
Verapamil	+	**455.10 > 149.60**	30	3.40	40
		455.10 > 164.60	30	3.54	

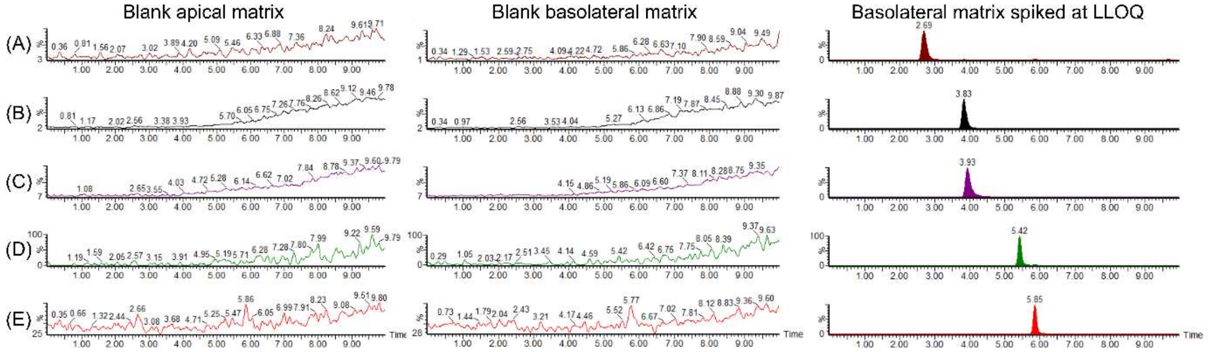

Figure S1. Chromatograms for the selectivity assessment of the UPLC–MS/MS method. Representative MRM chromatograms are shown for: the blank apical matrix (left column), the blank basolateral matrix (middle column), and the basolateral matrix spiked at the lower limit of quantification (LLOQ) for each analyte (right column). The rows correspond to: (A) Atenolol; (B) internal standard, Metoprolol; (C) Quinidine; (D) Propranolol and (E) Verapamil.

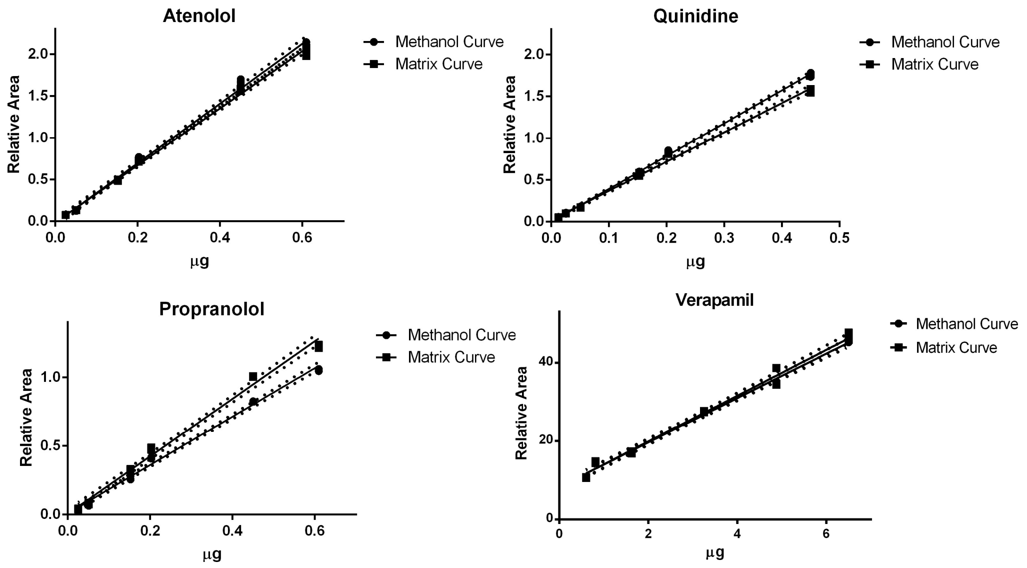

Figure S2. Comparison of calibration curves prepared in methanol and matrix for atenolol, propranolol, quinidine, and verapamil.

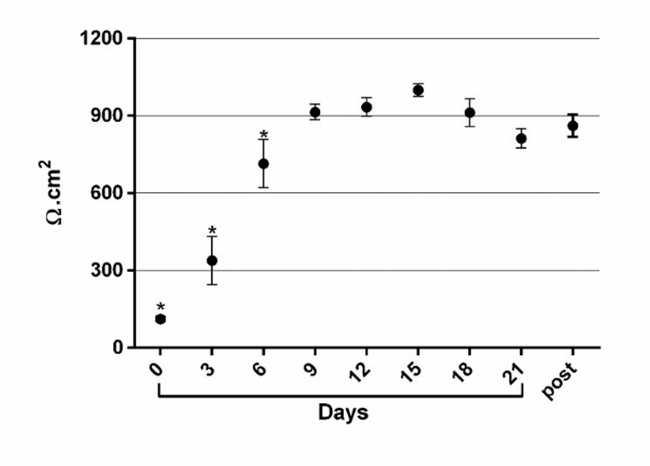

Figure S3. Transepithelial electrical resistance (TEER) measurements of Caco-2 cells cultured on Transwell™ permeable supports for 21 days. Results are expressed as mean ± standard deviation (SD). Data were analyzed using one-way analysis of variance (ANOVA) followed by Tukey's post hoc test. Values marked with * indicate statistically significant differences between groups, as determined by Tukey's post hoc test ($p < 0.05$).

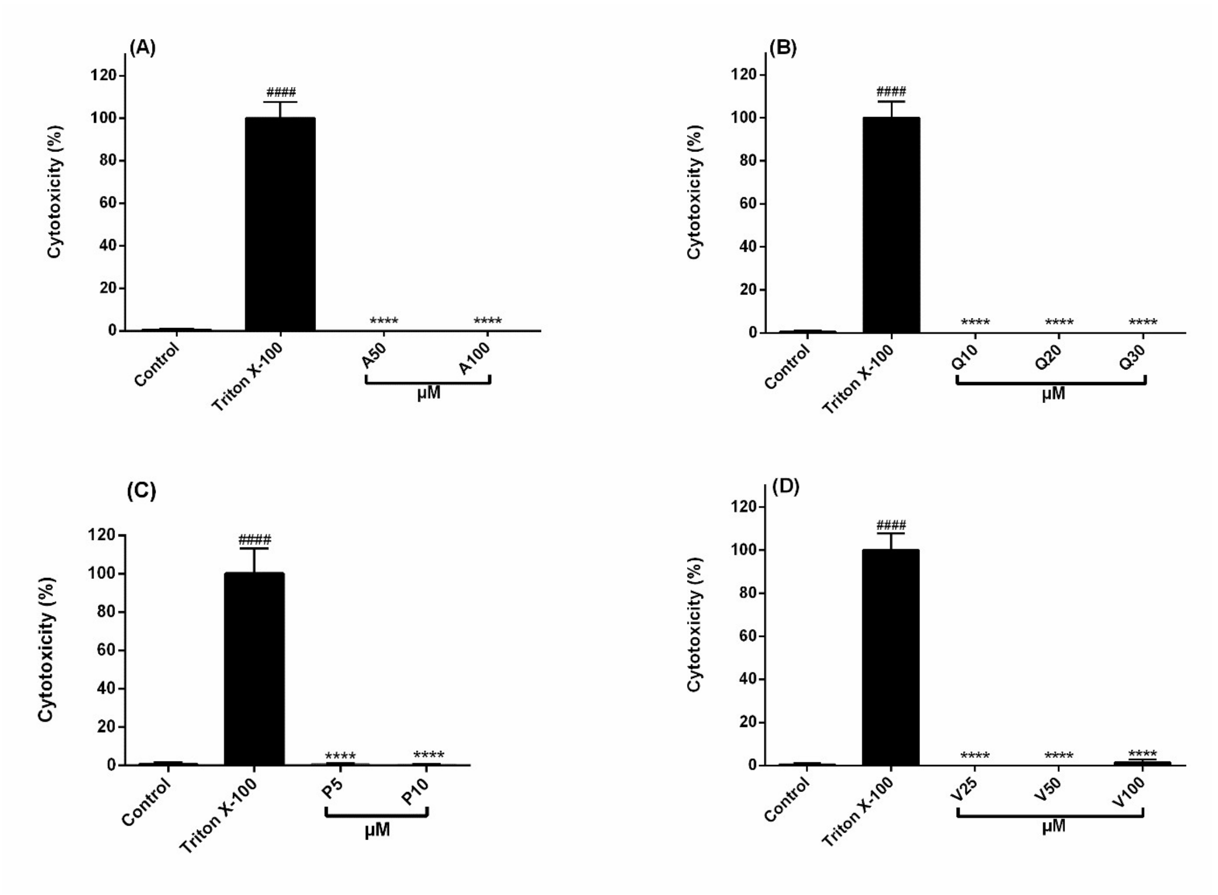

Figure S4. Cytotoxicity analysis of different compounds in Caco-2 cell culture. (A) atenolol (A50 and A100 µM), (B) quinidine (Q10, Q20, and Q30 µM), (C) propranolol (P5 and P10 µM), and (D) verapamil (V25, V50, and V100 µM). Triton X-100 (2%) was used as the positive control. Statistical significance: ####$p < 0.0001$ vs. basal control; ****$p < 0.0001$ vs. positive control (Triton X-100). HBSS with 1% DMSO was used as the basal control.

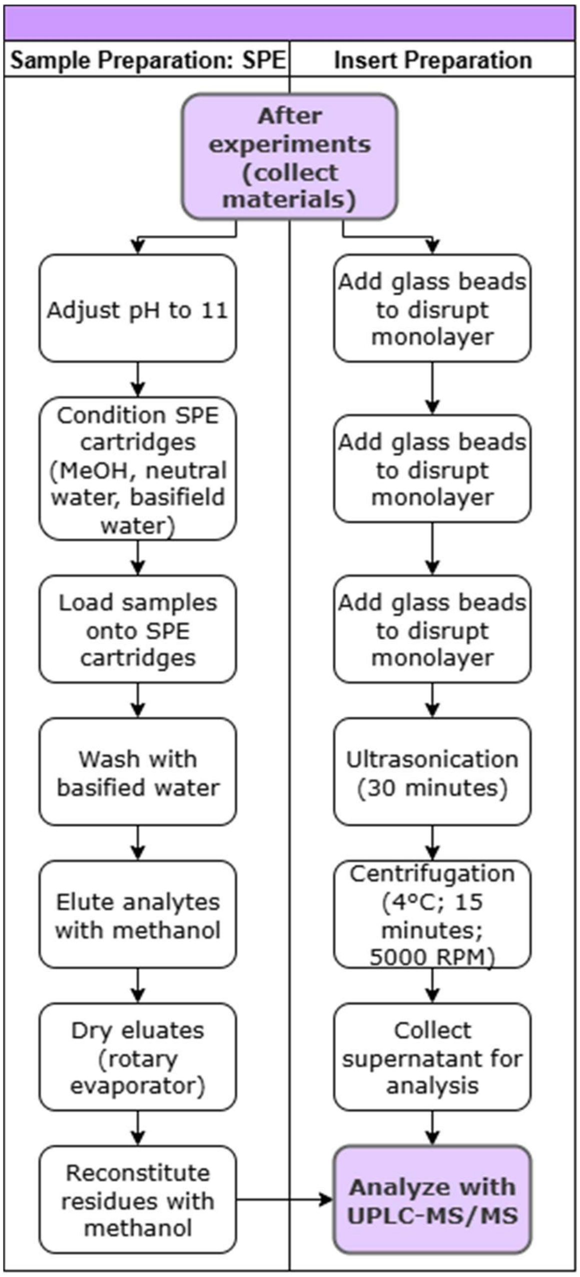

Figure S5. Workflow diagram illustrating the step-by-step procedure for sample preparation.
